# Two new records from China: *Aphaereta* (*Atopandrium*) *debilitata* Morley and *Elasmosoma berolinense* Ruthe (Hymenoptera, Braconidae)

**DOI:** 10.3897/BDJ.14.e181750

**Published:** 2026-03-25

**Authors:** Zibaidam Amat, Cornelis Van Achterberg, Guo-Shuai Zhao, Jia-Chen Zhu, Pu Tang, Qin Li

**Affiliations:** 1 Xinjiang University, Xinjiang, China Xinjiang University Xinjiang China; 2 Zhejiang University, Hangzhou, China Zhejiang University Hangzhou China; 3 Zhejiang A&F University, Hangzhou, China Zhejiang A&F University Hangzhou China

**Keywords:** Alysiinae, Euphorinae, Xinjiang

## Abstract

**Background:**

The genera *Aphaereta *Förster, 1863 (tribe Alysiini) and *Elasmosoma *Ruthe, 1858 (tribe Neoneurini, subfamily Euphorinae) represent two distinct and ecologically specialised lineages within the family Braconidae. *Aphaereta *is a cosmopolitan genus with approximately 48 described species worldwide, with four species recorded in China. *Elasmosoma *is a small genus comprising 13 described species globally, two of which are recorded in China. Given the limitations of existing faunistic surveys, it is inferred that a substantial number of species within these groups likely remain undiscovered in China, particularly in its north-western regions.

**New information:**

This study reports first records of two parasitoid wasp genera *Aphaereta* and *Elasmosoma* in the Xinjiang Region of China. Specimens were collected in 2024 using Malaise traps in apple, apricot and pear orchards in the Aksu Prefecture. Amongst these, *Aphaereta debilitata* and subgenus *Atopandrium* are recorded from China for the first time.

## Introduction

The braconid genera *Aphaereta* and *Elasmosoma *are poorly represented in China, with only four and two previously-recorded species, respectively (Yu et al. 2016).

This paper documents the first Chinese records of *Aphaereta *(*Atopandrium*)* debilitata* Morley, 1933 and *Elasmosoma berolinense* Ruthe, 1858, thereby increasing the known species richness of these two genera in the country.

## Materials and methods

The specimens used in this study were collected by using Malaise traps in multiple orchards, in Aksu Prefecture, Xinjiang. For the identification of the specimen, a ZEISS STEMI305 binocular microscope was used. The images were captured using a Keyence VHX – 7100 camera and, for post-processing work, Adobe Photoshop 2021 and Adobe Illustrator 2023 software were used. The specimens are preserved in the Insect Specimen Collection of Zhejiang University.

For the morphological terms and the identification of the subfamilies, see van Achterberg (1993) and for identification of *Elasmosoma,* the key in Shaw (1985) and for *Aphaereta debilitata*, the re-description and illustrations by van Achterberg (1995) were used.

## Taxon treatments

### Aphaereta (Atopandrium) debilitata

Morley, 1933

6C8C119D-D44F-533A-A751-7879D450C466

#### Materials

- **Type status:** Other material. **Occurrence:** occurrenceRemarks: in apricot orchard; sex: 3 female; occurrenceID: BE85C173-B1C4-5911-8D1F-500B821BB7CC; **Location:** country: China; countryCode: Xinjiang; stateProvince: Aksu; **Event:** samplingProtocol: Malaise trap; year: 2024; month: Apr
- **Type status:** Other material. **Occurrence:** occurrenceRemarks: in apricot orchard; sex: 1 female; occurrenceID: 803A9372-A57D-5E97-A3CE-F9EA8DD71B4B; **Location:** country: China; countryCode: Xinjiang; stateProvince: Aksu; **Event:** samplingEffort: Malaise trap; year: 2024; month: Sep

#### Description

**Figure 1.**
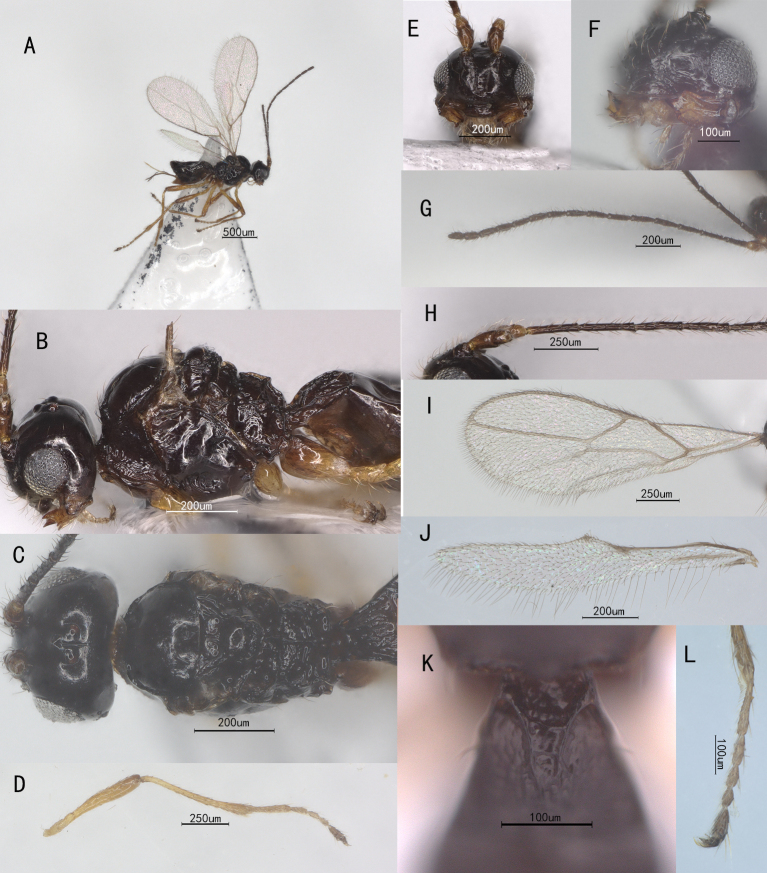
*Aphaereta (Atopandrium) debilitata* Morley, ♀, China. **A** body, lateral aspect; **B** Mesosoma, lateral aspect; **C** Mesosoma, dorsal aspect; **D** Hind leg, lateral aspect; **E** Head, anterior aspect; **F** Head, lateral aspect; **G – H** Antenna, lateral aspect; **I – J** Wing; **K** First tergite, dorsal aspect; **L** Hind tarsus, lateral aspect.

**Head**. Antennomeres 17, antenna 1.1× as long as forewing, 1.3× as long as body and 2.3× as long as head and mesosoma combined; first flagellomere 0.8× as long as second flagellomere; first and second flagellomeres 3.1× and 5.6× as long as wide, respectively; fourth flagellomere 3.0× as long as wide, 1.5× third flagellomere, 1.4× fifth flagellomere (Fig. 1G and H); maxillary palp with 4 segments, labial palp with 2 segments; temples roundly parallel-sided behind eyes; ocelli medium-sized, almost in circular formation, short longitudinal sulcus extends from ocellar region posteriorly to occiput, OOL: OD: POL = 7 : 2 : 3; frons largely smooth, rugose and with shallow depression in front of median ocellus; vertex smooth and shiny; face 2.7× wider than high, sparsely with long setae, smooth; clypeus smooth, 3.3× wider than high, 2× as wide as face, slightly curved ventrally; mandibles widened towards apex, its first tooth shorter than second tooth; middle tooth wide basally and narrowed towards apex, rounded apically (Fig. 1E and F). 

**Mesosoma**. Length of mesosoma 1.4× its height; side of pronotum rugose; propleuron smooth, but pronotum coarse sculptured; mesoscutum entirely smooth and glossy, anteriorly rounded, with both the anterior and lateral margins upturned and punctate; notauli only anteriorly present; medio-posterior depression absent; mesopleuron smooth, precoxal sulcus short and broad; scutellum smooth; propodeum bears a median longitudinal carina and regular transverse ridges (Fig. 1B and C).

**Metasoma**. First tergite 1.4× longer than its maximum width, apically 1.8× wider than its minimum width, largely rugulose basal carinae gradually converging to form a V-shaped area (Fig. 1K); dorsope present, remainder of metasoma smooth and glossy; ovipositor 0.6× as long as first tergite.

**Wings**. Wings hyaline, surface and margins setose; pterostigma short and narrow, almost merged with vein R1; fore-wing (Fig. 1I), first and second submarginal cells and first discal cell fused (veins 1-SR+M and 2-SR absent); vein 2-1A absent. Hind-wing (Fig. 1J): veins cu-a and r-m absent.

**Legs**. Hind tibia 11.2× as long as wide, slender; hind femur 5.2× as long as wide (Fig. 1D); basitarsus 0.8× as long as following tarsomeres (Fig. 1L).

**Colour**. Body black to chocolate brown; scape and pedicel of antenna yellowish; wing veins and pterostigma brown; legs yellowish, tarsal bases approaching body colour.

#### Distribution

Austria; former Czechoslovakia; Germany; Hungary; Japan; Netherlands; Poland; Russia; Spain; United Kingdom (Yu et al. 2016) and China (new record).

#### Biology

Parasitoid of *Scatella stagnalis * (Fallén, 1813), see Goto and Maetô (2000).

#### Notes

We use for *Aphaereta debilitata* Morley, 1933 the subgenus *Atopandrium* Graham, 1952, considering the aberrant wings of male and female. The species and subgenus are recorded from China for the first time.

### Elasmosoma berolinense

Ruthe, 1858

1BD4124B-21F0-5103-9708-F0CC353D9844

#### Materials

- **Type status:** Other material. **Occurrence:** sex: 1 female; occurrenceID: 59EC96FE-FF0E-5620-B507-94511B11D7A5; **Location:** country: china; countryCode: Xin Jiang; stateProvince: Aksu; **Event:** samplingProtocol: pear orchard - Malaise trap; year: 2024; month: Apr

#### Description

**Figure 2.**
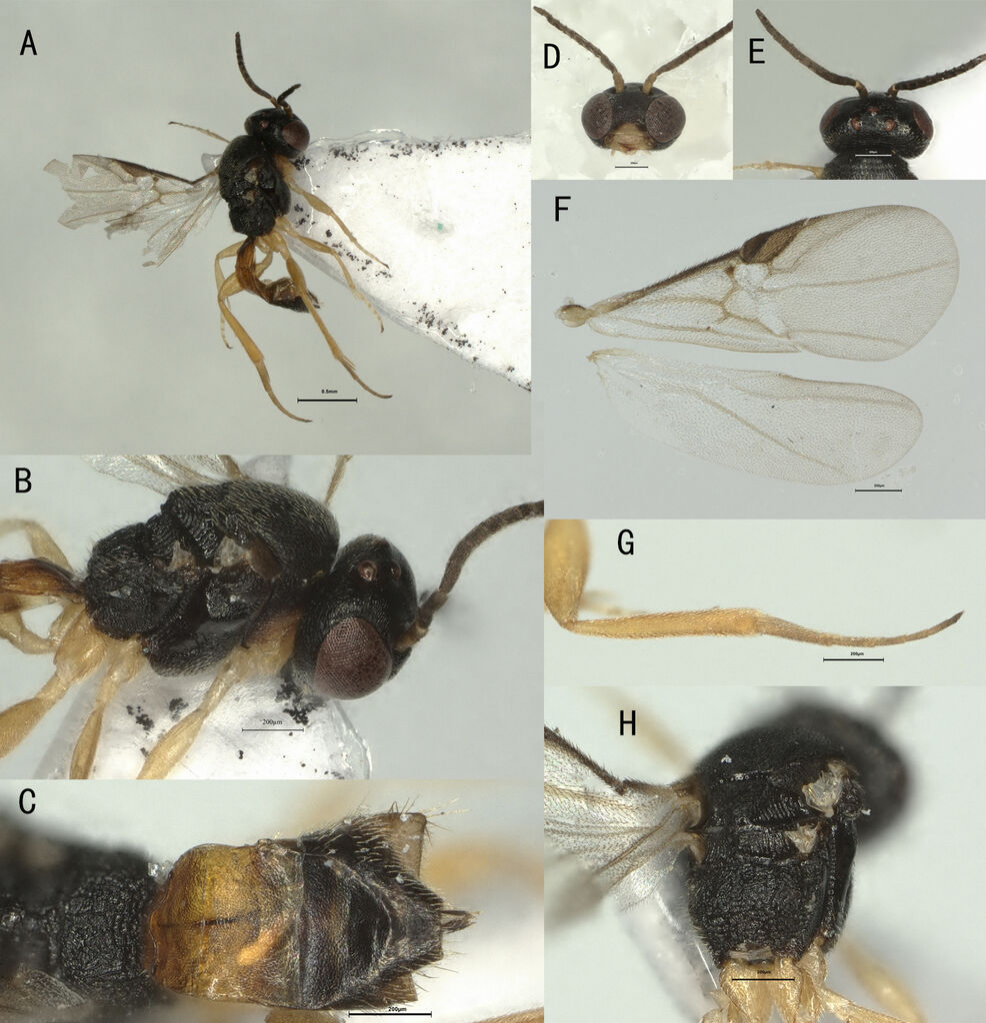
*Elasmosoma berolinense* Ruthe, ♀, China. **A** Body, lateral aspect; **B** Mesosoma, lateral aspect; **C** Metasoma, dorsal aspect; **D** Head, anterior aspect; **E** Head, dorsal aspect; **F** Wings; **G** Hind leg, lateral aspect; **H** Propodeum, dorsal aspect.

**Head**. Antennomeres 13, antenna 0.6× as long as fore-wing, 0.4× as long as body, and 1.3× as long as head and mesosoma combined; first flagellomere 1.4× as long as second flagellomere; first and second flagellomeres 1.3× and 1.2× as long as wide, respectively; maxillary palp with 2 segments, labial palp with 1 segment; temples roundly narrowed behind eyes; ocelli medium-sized, almost in right triangle, OOL: OD: POL = 9: 5: 13; frons largely punctate, rugose in front of median ocellus; vertex punctate-striate; face as wide as high, sparsely setose, strigose; clypeus rugulose, 3.4× wider than high, 1.2× as wide as face, ventral margin concave medially; mandibles stout, straight, its first tooth much longer than second tooth and very acute (Fig. 2D–E).

**Mesosoma**. Length of mesosoma 1.3× its height; side of pronotum coriaceous (Fig. 2B); propleuron punctate-rugose; mesopleuron dorsally rugose, ventrally largely rugulose; prepectal carina present; episternal scrobe short, wide and deep; precoxal sulcus deep and wide; mesonotum densely setose, flat, coriaceous; notauli absent; scutellar sulcus deep, crenulate; scutellum convex, punctate-rugose; propodeum reticulate-rugose and with regular carina-like rugae (Fig. 2H).

**Metasoma**. First tergite 1.3× longer than its maximum width, apically 2× wider than its minimum width, largely rugulose; second and third tergites granulate-rugulose; first tergite with laterope, remaining segments smooth or nearly so, rather compressed and glossy; hypopygium with “prong-like” structures because of deep medial emargination, setae along apical margin long (Fig. 2A–C), especially laterally; ovipositor short; ovipositor sheath robust and short, 1.8× as long as wide (Fig. 2C).

**Wings**. Fore-wing (Fig. 2F): veins pigmented; 1-R1 longer than pterostigma; vein r issued in front of middle of pterostigma; 1-M short, 0.6× as long as r; cu-a oblique and distinctly longer than 1-CU1, cu-a:1-CU1 = 6:3. Hind wing (Fig. 2F): venation extremely reduced, without closed cells.

**Legs.** Fore tibia 3.3× as long as wide; fore tibial spur 1.1× as long as basitarsus; Middle leg normal, tibia 3.4× as long as wide; middle tibial spurs straight. Hind tibia 6.7× as long as wide (Fig. 2G).

**Colour**. Mainly black; fore-wings slightly darkened, veins light brown; fore leg, middle leg and hind femur pale yellowish, hind tibia and tarsus dark brown; ovipositor yellowish; antenna (except scape and pedicel) and pterostigma dark brown; first tergite (apically), second and third tergites yellow.

#### Distribution

Albania, Austria, Bulgaria, Croatia, Denmark, Finland, France, Germany, Greece, Hungary, Iran, Italy, Kazakhstan, Macedonia, Moldova, Mongolia, Netherlands, Poland, Russia (Amur Oblast, Krasnodar Kray, Primor'ye Kray), Slovakia, Slovenia, Sweden, Tajikistan, Turkiye, United Kingdom, Japan (main islands) (Yu et al. 2016) and China (new record).

#### Biology

Parasitoid of *Formica *spp. (incl.* F. fusca*, *F. rufa*, *F. rufa japonica*, *F. pratensis*, *F. sanguinea*) and *Lasius niger *Linnaeus, 1758 (see Huddleston (1976)); *Camponotus vagus* Scopoli, 1763 (see Panis (2007)).

#### Notes

**Figure 3.**
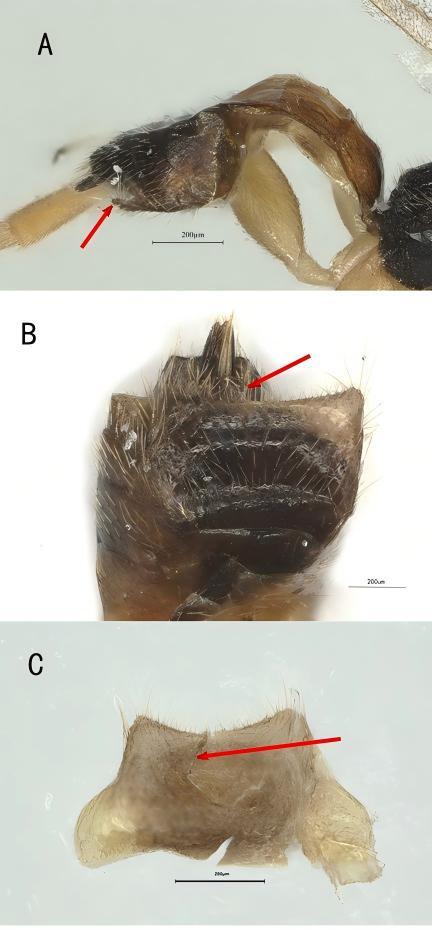
*Elasmosoma berolinense* Ruthe, ♀, China. **A** Metasoma, lateral aspect; **B** Apical part of metasoma, ventral aspect; **C** Hypopygium, ventral aspect.

This study reports the first record of *Elasmosoma berolinense* Ruthe, 1858 in China. The identification of this species is primarily based on the morphological characteristics of the female hypopygium: the presence of the unique apical "prong-like" structures (Fig. 3C) fully corresponds to the morphology illustrated in fig. 20 of *E. berolinense* in van Achterberg and Koponen (2003). In the initial draft, the specimen was tentatively identified as *E. luxemburgense* because the hypopygium was retracted. However, after dissection, the morphology of its hypopygium became more clear (Fig. 3C) and without doubt belongs to *E. berolinense*. Herewith, this study confirms the first record of *E. berolinense* in China.

## Supplementary Material

XML Treatment for Aphaereta (Atopandrium) debilitata

XML Treatment for Elasmosoma berolinense
